# Structure of the Hepatitis B virus capsid quasi-6-fold with a trapped C-terminal domain reveals capsid movements associated with domain exit

**DOI:** 10.1016/j.jbc.2023.105104

**Published:** 2023-07-28

**Authors:** Christine Kim, Christopher J. Schlicksup, Carolina Pérez-Segura, Jodi A. Hadden-Perilla, Joseph Che-Yen Wang, Adam Zlotnick

**Affiliations:** 1 Molecular and Cellular Biochemistry Department, Indiana University, Bloomington, Indiana, USA; 2 Department of Chemistry and Biochemistry, University of Delaware, Newark, Delaware, USA; 3 Department of Microbiology & Immunology, Pennsylvania State University College of Medicine, Hershey, Pennsylvania, USA

**Keywords:** HBV, self-assembly, capsid, nuclear localization signal, structural virology, physical virology

## Abstract

Many viruses undergo transient conformational change to surveil their environments for receptors and host factors. In Hepatitis B virus (HBV) infection, after the virus enters the cell, it is transported to the nucleus by interaction of the HBV capsid with an importin α/β complex. The interaction between virus and importins is mediated by nuclear localization signals on the capsid protein’s C-terminal domain (CTD). However, CTDs are located inside the capsid. In this study, we asked where does a CTD exit the capsid, are all quasi-equivalent CTDs created equal, and does the capsid structure deform to facilitate CTD egress from the capsid? Here, we used Impβ as a tool to trap transiently exposed CTDs and examined this complex by cryo-electron microscopy. We examined an asymmetric reconstruction of a T = 4 icosahedral capsid and a focused reconstruction of a quasi-6-fold vertex (3.8 and 4.0 Å resolution, respectively). Both approaches showed that a subset of CTDs extended through a pore in the center of the quasi-6-fold complex. CTD egress was accompanied by enlargement of the pore and subtle changes in quaternary and tertiary structure of the quasi-6-fold. When compared to molecular dynamics simulations, structural changes were within the normal range of capsid flexibility. Although pore diameter was enlarged in the Impβ-bound reconstruction, simulations indicate that CTD egress does not exclusively depend on enlarged pores. In summary, we find that HBV surveillance of its environment by transient exposure of its CTD requires only modest conformational change of the capsid.

Like many viruses, the Hepatitis B virus (HBV) replication cycle is dependent on its icosahedral nucleocapsid to protect its genome, deliver it to the appropriate cellular compartment, and release the genome. The HBV core (or capsid) protein (Cp) is 183 residues-long with an all-helix N-terminal assembly domain (residues 1–149) and the C-terminal nucleic acid-binding domain (residues 150–183). Purified full-length Cp is often called Cp183 and purified assembly domain, Cp149. The C-terminal domain (CTD) of each Cp plays multiple essential roles during the HBV life cycle. These 34 residues are arginine-rich, localized to the capsid interior, and thus interact with viral RNA or DNA during virus assembly and reverse transcription (1). CTD organization can be influenced by phosphorylation or the presence of internal genome (2, 3, 4). Remarkably, CTDs periodically protrude through the capsid to interact with host proteins, including various kinases (5, 6, 7).

During HBV infection, assembly may result in empty capsids (∼90%) or RNA-filled capsids (∼10%) (8, 9). RNA-filled capsids contain a terminally redundant RNA copy of the genome and a copy of viral reverse transcriptase. RNA-filled capsids mature by reverse transcription of the linear ssRNA to a circular, largely double-stranded DNA (10, 11). Notably, empty capsids are hyper-phosphorylated, and DNA-containing capsids are hypo-phosphorylated (12, 13). DNA-containing and empty HBV capsids are trafficked to the nucleus by a complex of importins α and β (Impα and Impβ) to deposit the viral genome or core protein (14, 15, 16, 17, 18). The CTD carries one or more nuclear localization signals (19). However, CTDs can bind to Impβ alone *in vitro*, making this host protein useful for studying CTD dynamics (4). The Impβ family are ∼96 kDa proteins composed of 18 to 19 HEAT domains arranged in a spiral (20); Impβ canonically binds to a ∼40 residue highly basic importin beta binding domain that is apparently emulated by the HBV CTD.

Cp forms a stable homodimer, where 90 or 120 Cp dimers assemble to form T = 3 or T = 4 icosahedral capsids, respectively, with the T = 4 complex the predominant (∼95%) assembly product (21). In a T = 4 capsid, the Cp monomers are designated as A, B, C, and D, also described as AB and CD dimers. The icosahedral T = 4 HBV capsid has 5-fold, and quasi-6-fold (icosahedral 2-fold) vertices, where the 5-fold is composed of 5 A subunits and the quasi-6-fold has a B-C-D-B-C-D repeat (Fig. S1). Although the Cp sequence is identical, the monomers adapt to slightly different quasi-equivalent environments within the capsid structure (22). In dimers, helices 3 to 4 from each subunit form a four-helix bundle, the intradimer dimer interface (Fig. S1); these comprise the spikes that punctuate the capsid surface (23). In capsids, the interdimer interface is formed by helix 5 from one dimer, filling a groove between helix 5 and the spike region of an adjacent dimer (21, 24). The interdimer interface appears to be a flexible contact, whereas dimers have limited flexibility (25, 26).

Low resolution structures of empty capsids bound to Impβ indicated that Impβ binds near the quasi-6-fold symmetry axis, and the overall capsid appeared to generally maintain T = 4 geometry (27). Nonetheless, a significant amount of Impβ was internalized during those experiments, suggesting transient capsid disruption. Due to the resolution of the structures and icosahedral averaging, none of the reconstructions revealed where the CTDs exited to interact with Impβ or show subtle conformational changes to the capsid. Here, we used different sample preparation and cryo-EM reconstruction strategies to answer these questions. We observed that Impβ-bound capsids (at 3.8 Å and 4.2 Å resolution) retained icosahedral geometry, even when no symmetry was imposed during computation. Focused reconstructions (at 4.4 Å resolution) of quasi-6-fold complexes indicate that the central pores were expanded when occupied by Impβ, and CTDs of a subset of quasi-equivalent Cps exit the pore.

## Results

### Sample preparation and cryo-EM reconstruction strategies

To examine the exit path of the CTD and Impβ-induced conformational changes to the capsid, two sample preparation and cryo-EM reconstruction strategies were performed (Fig. S1). Empty capsids at 11.9 μM Cp183 dimer were mixed with either 8.0 μM or 20.3 μM Impβ (1:80 or 1:205 capsid to Impβ ratios, respectively). For simplicity, we will refer to the 1:80 and 1:205 ratios as the “low” and “high” Impβ ratios, respectively. Previously, the low Impβ ratio was used to reconstruct the heterocomplex (27). The density maps from those samples suggested that the 30 quasi-6-fold sites were partially occupied or that Impβ was extremely mobile. Pursuing higher occupancy of the quasi-6-fold vertices was stymied because high concentrations of Impβ led to capsid deformation (27). For this study, we tested the two ratios in order to work with a condition that minimized capsid damage and another condition that maximized saturation.

### Impβ-bound capsids maintain icosahedral symmetry

**Figure 1:**
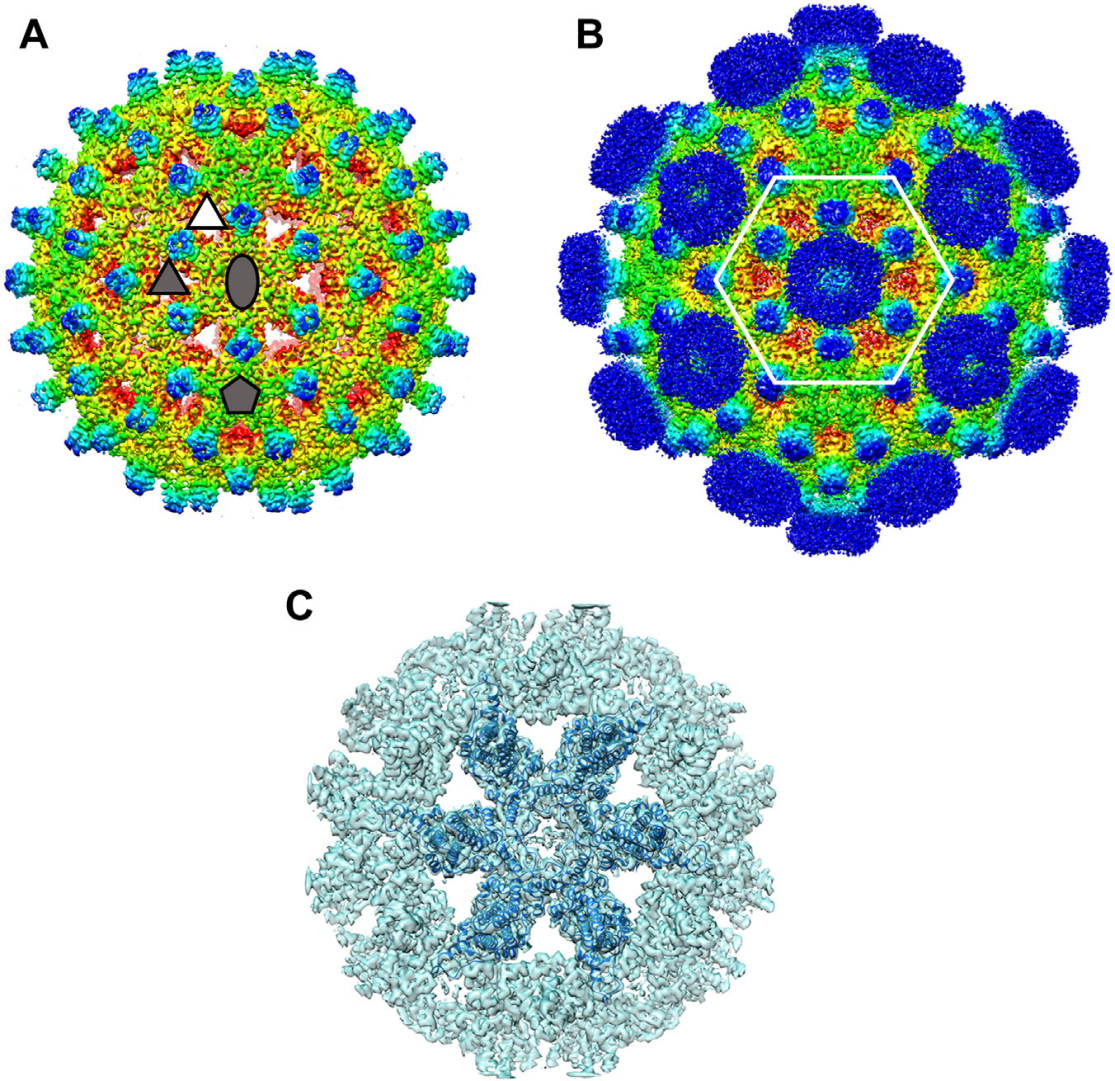
**Cryo-EM reconstruction of Impβ–bound empty capsids shows density at quasi-6-folds.***A*, for this reconstruction, we used 2D class averages (Fig. S2) that had capsids which both appeared to be empty capsids and Impβ density (29,006 particles). These were used for this icosahedrally averaged reconstruction determined to 4.2 Å resolution. By showing strong density (contoured to 2.8σ), we see only the HBV capsid protein. A subset of symmetry operators that lie on the equator are shown in *dark gray*: 5-fold (*pentagon*), 3-fold (*triangle*), and 2-fold (quasi-6-fold, *oval*). A quasi-3-fold is identified by a *white triangle*. *B*, density for Impβ was visible when the contour level of the map was decreased to 1.3σ. This density forms disordered clouds above the quasi-6-fold vertex. A quasi-6-fold (icosahedral 2-fold) is outlined in *white*, indicating the area highlighted in local reconstructions. Density in in *panels A* and *B* is colored based on radius, with *blue* at >175 Å and *red* at <100 Å. *C*, density (*cyan*) for a local reconstruction of a quasi-6-fold vertex, determined to 4.0 Å resolution. Shown in density is a flexibly fit model of six core protein dimers. In this orientation, the A subunits on the extreme *right* and *left*.

Initially, we examined Impβ complexes, where *in vitro* assembled empty capsids were mixed with Impβ at the high Impβ condition. First, Cp183 capsids were assembled in high ionic strength to allow assembly and overcome the electrostatic repulsion of CTDs clustered inside the capsid at 5-fold and quasi-6-fold vertices (2). After addition of Impβ to these capsids, the mixture was dialyzed overnight into a low salt buffer to allow electrostatic binding and then samples were subjected to cryo-EM. Under high Impβ conditions, many capsids are damaged (27). From the micrographs, capsid particles were picked and then sorted based on their morphology *via* 2D classification to remove damaged particles. Several rounds of 2D classification were performed to enrich for T = 4 particles that appeared empty (*i.e.*, clear) and had Impβ protein densities (Fig. S2). The final round of 2D classification yielded 20 classes (Fig. S2*B*). All 20 2D class averages revealed spherical and intact capsids. Eighteen classes clearly showed weak but distinct Impβ density, decorating the capsid exterior, compared to previous experience with undecorated capsids. Particles from these 18 classes were pooled and used to produce an initial model (with C1 symmetry), which was subsequently used to generate a refined structure with icosahedral (I2) symmetry. The postprocessed I2 density map (4.2 Å) showed capsids with the expected T = 4 geometry (Fig. 1*A*). Consistent with previous cryo-EM structures (27), when contoured to show weaker density, the isosurface map showed highly disordered densities of Impβ extending from the center of the quasi-6-fold symmetry axes (Fig. 1*B*). The shape of the Impβ density suggested to us that the bound host protein is sampling multiple orientations above the quasi-6-fold, resulting in the observed a funnel-like density.

Similarly, under low Impβ conditions where capsids were less damaged by Impβ, we collected cryo-EM data for a C1 asymmetric reconstruction (3.8 Å), with the hypothesis that this approach would capture capsid deformation. We observed an icosahedral map which showed heterogenous rings of importin density when contoured at 1σ (Fig. S3, *A* and *B*). At regions where the Impβ density is thicker, we observed a thin, rope-like density emerge from the center of the quasi-6-fold and connect to the Impβ density (Fig. S3*C*). This is consistent with the hypothesis that CTDs may exit the central pore. As observed in the I2 map (Fig. 1*B*), the diffuse density indicated that the Impβ protein is adopting various orientations and possibly conformations. Though we had not imposed icosahedral symmetry, we could not exclude the likelihood that irregular capsids combined together would result in *de facto* icosahedral averaging.

### Focused reconstruction of hexamers indicates that Impβ trap capsids in an expanded state

To examine structural changes of the Impβ-capsid heterocomplex, focused reconstruction of the quasi-6-fold regions (also referred to as hexamers) of the capsid was implemented. We had originally predicted that Impβ-binding to CTDs would break quasi-6-fold symmetry, leading to asymmetric hexamers. Although, the C1 and I2 capsid reconstructions showed that capsids appeared to retain their icosahedral geometry, these structures were averages of numerous particles. It was possible that the averaging dampened features of the capsid where symmetry was broken, and we wanted to investigate if focused reconstruction could tease out asymmetry. To perform focused reconstruction, we used the particle coordinates of the I2 density map from the high Impβ sample since these complexes were geometrically defined and biochemically had the best chances of having their quasi-6-fold sites occupied. The capsid particle coordinates were symmetry expanded so that all 30 hexamers were independently included. These hexamers were used to reconstruct an initial hexamer model (C1 symmetry). The resulting structure retained the 2-fold symmetry of a T = 4 quasi-6-fold for Cp with the disordered Impβ density emerging from the center of the hexamer (Fig. S4). Subsequent 3D classification was performed with C1 symmetry to group hexamers based on differences in morphology. We generated 10 3D class averages, six of which were well resolved and also appeared to be 2-fold symmetric. In the other three classes, the secondary and tertiary structures were unclear.

The six “symmetric” classes were pooled together to produce a 4.0 Å hexamer map (Figs. 1*C* and S4). A hexamer model was flexibly fit into the final density map. In subsequent examination, this structure was compared to a hexamer from a Cp183 capsid (pdb: 3J2V) (28). This apo structure was chosen because it does not have Impβ and was filled with RNA to tie up free CTDs. As our structure is only at 4 Å resolution, we emphasize our structural comparisons on groups of amino acids or units of secondary structure to absorb error that might arise in single residue comparisons. To elucidate changes to secondary and tertiary structure, the flexibly fit A, B, C, and D monomers were separately superimposed on their prospective apo models (Fig. S5). We observed no systematic structural difference; a residue-by-residue comparison shows little deviation excepting a few residues, particularly in the spike region (Fig. S5). The RMSD for the A, B, C, and D monomers were 1.09, 0.86, 0.84, and 1.0 Å, respectively, a modest difference at 4 Å resolution.

**Figure 2:**
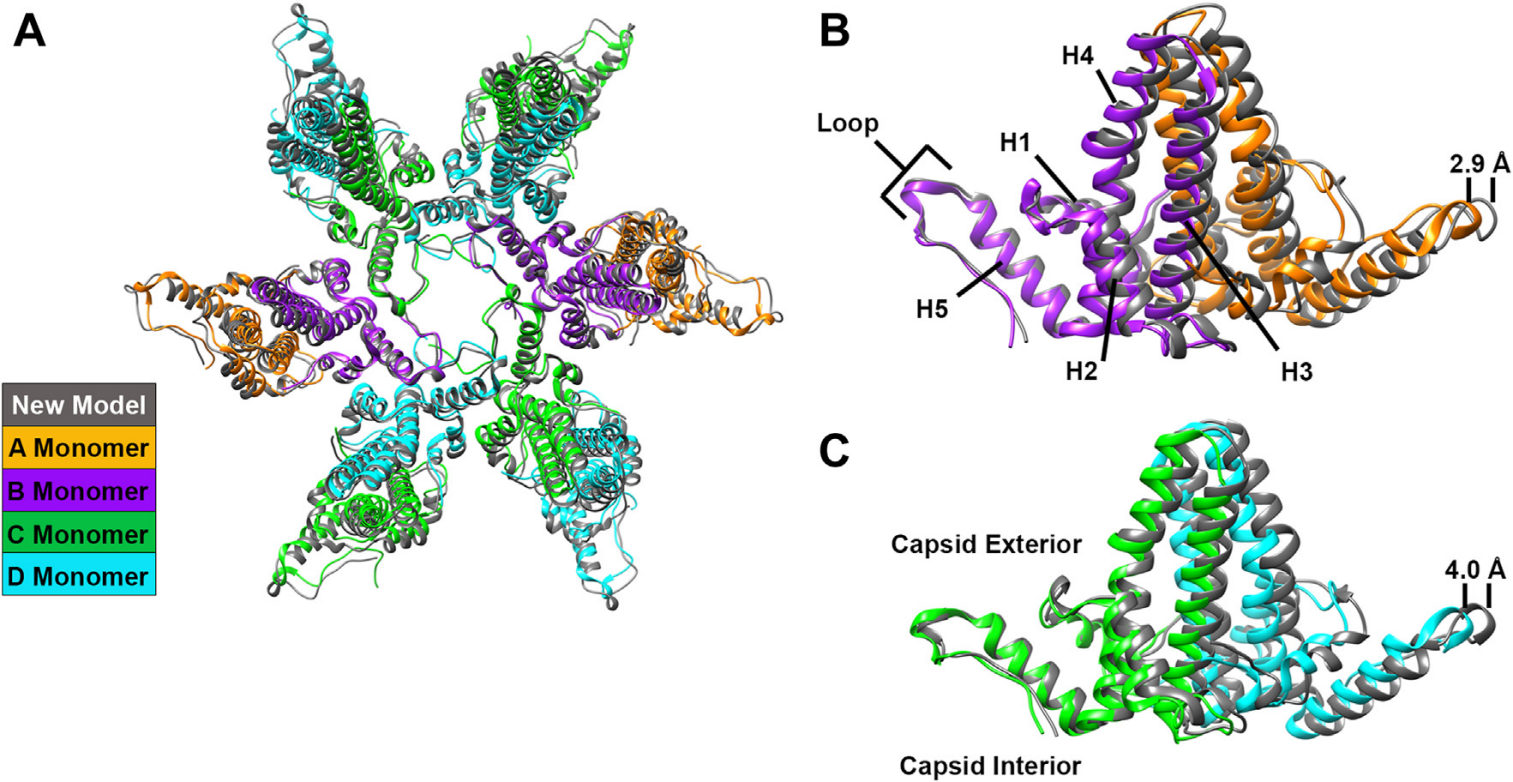
**Superposition of molecular models from a flexibly fit hexamer and from an RNA-filled Cp183 capsid show that the quasi-6-fold vertex is systematically distorted concomitant with binding Impβ.***A*, the molecule model flexibly fit into the quasi-6-fold density (*gray*, shown in Fig. 1*C*) is superpositioned on an apo-hexamer from a Cp183 capsid (pdb: 3J2V). The color scheme is defined by the inset on *bottom right* of this figure. The alignment is closest at the *center* of the quasi-6-fold. *B* and *C*, a sideview of the superpositioned dimers from *panel A* with the quasi-6-fold axis on the *left*; displacement of dimers (after Impβ-binding) has been labeled on the *right*. *B*, the helices in the assembly domain are identified in subunit ‘B’. At the leftmost edge of the B subunit, the loops at the end of helix 5 are closely aligned. At the other end of the dimer, the A subunit shows a substantial offset of the loop at the end of helix 5 (see Fig. S1*B* for a labeled map of a dimer). *C*, a CD dimer superposition where the C subunit on the *left* is proximal to the center of the quasi-6-fold, and the D subunit is distal to it. As with AB dimer, the distal D subunit shows a substantial offset. *Panel C* also indicates the capsid interior and exterior relative to the dimer.

Unlike the tertiary structure of individual subunits, the six Cp183 dimers making a hexamer show a considerable change in quaternary structure compared to the 3J2V apo model (Figs. 2*A* and S6). In the overall superposition, the loops surrounding the quasi-6-fold match closely. The loops in question, residues 130 to 135, immediately follow helix 5 which plays a central role in interdimer contacts. Distal to the pore, the equivalent loops at the other end of each show a substantial displacement of 2.9 to 4.0 Å (Figs. 2*C* and S6).

**Figure 3:**
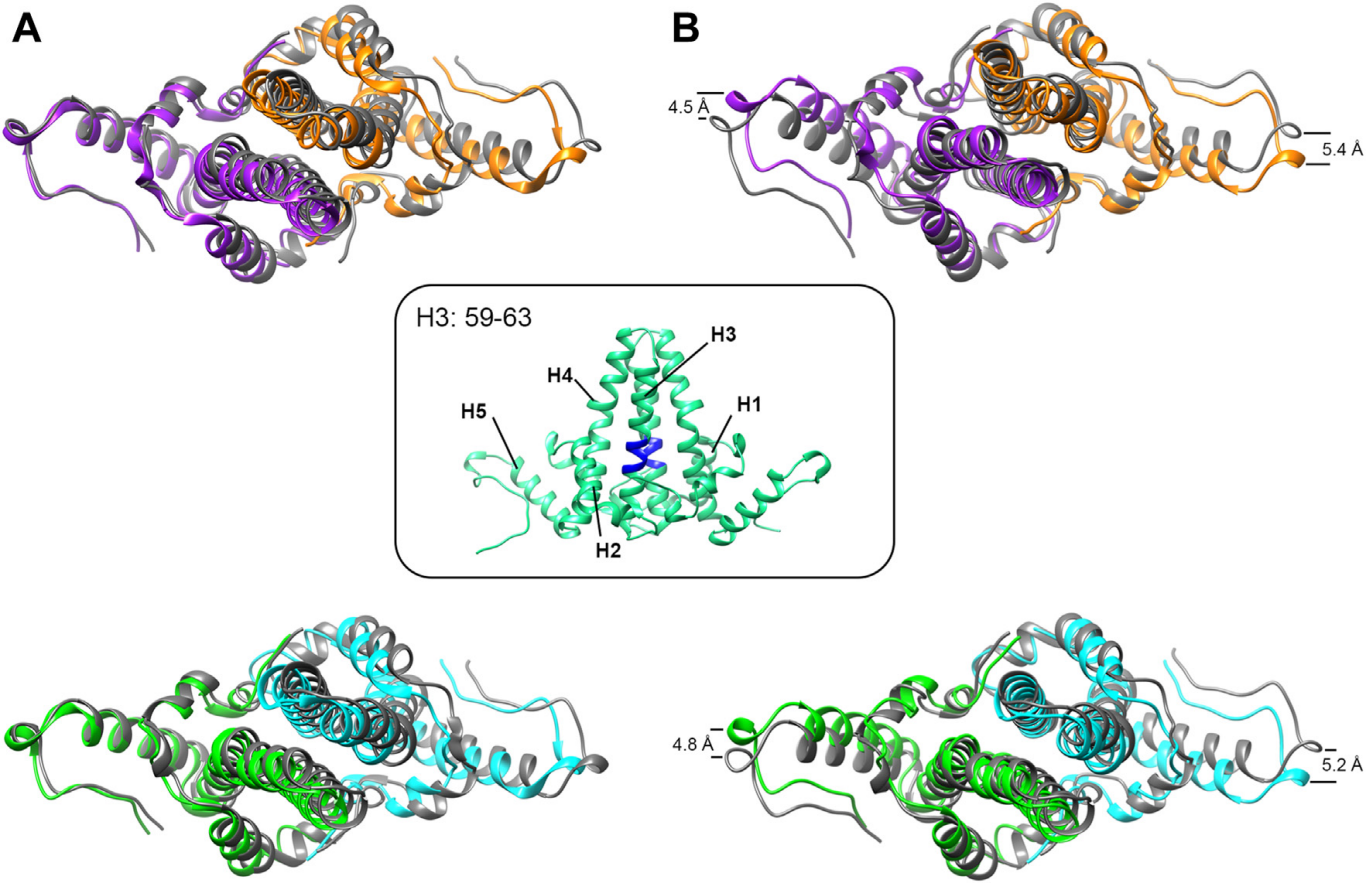
**Superposition of dimers show that hexamer and dimer quaternary structure are distorted.** We compare AB dimers (*top row*) and CD dimers (*bottom row*) with two different superpositioning strategies. *Column A*, dimers are super-positioned based the center of mass of the quasi-6-fold (as in Fig. 2, *B* and *C*, but here viewed from the *top*). For the subunit close to the quasi-6-fold, the loop at the end of helix 5 of the +Impβ model closely matches the loop from the RNA-filled capsid. *Column B*, when individual dimers are superpositioned based on helix 3 from the intradimer interface (see inset), the four helix bundles match well but the loop at the end of helix 5 for both subunits has moved a substantial distance. Displacements of each dimer were measured from A131 to A131 and ranged from 4.5 to 5.4 Å. This observation is consistent with the hypothesis that helix 5 is connected to a central “chassis” region by a flexible hinge. Subunits are colored as in Figure 2.

We found that quaternary structure changed at both interdimer contacts (Fig. 2*A*) and at intradimer contacts (Fig. 3*B*). When dimers were aligned on the basis of the two copies of helix 3 at the dimer interface (Fig. 3 inset), part of the four-helix bundle at the intradimer interface, we observe that helix 5 of each monomer has rotated so that the Cα for residue 131 in the loop is displaced by 4.5 to 5.3 Å (5.0 ± 0.4 Å). This motion is consistent with the observation that Cp structural elements are connected to the central structure by flexible hinges (here Gly111 is a likely pivot point) (29). However, it appears that other structural elements, including helix 4, have also moved, contributing to the change in dimer quaternary structure.

### The central pores of Impβ-bound hexamers are dilated

The change in hexamer tertiary and quaternary structure requires that the rest of the capsid adjust. Was this considerable motion imposed by binding Impβ or was it within the normal range of capsid movement and had just been trapped by Impβ? To address this question, we mined data from a 1-μs molecular dynamics (MD) simulation of an intact HBV capsid (26). We note that CTD exposure to proteases indicates a half-life on the order of minutes (2, 4), a timescale far beyond what is feasible for atomistic MD simulations. However, the simulation data provide important insights into the range of motions that are normal for the capsid at equilibrium. The simulated capsid model represents the assembly domain (Cp149), lacking the CTD, and exhibits a dramatic range of structural movement (26).

**Figure 4:**
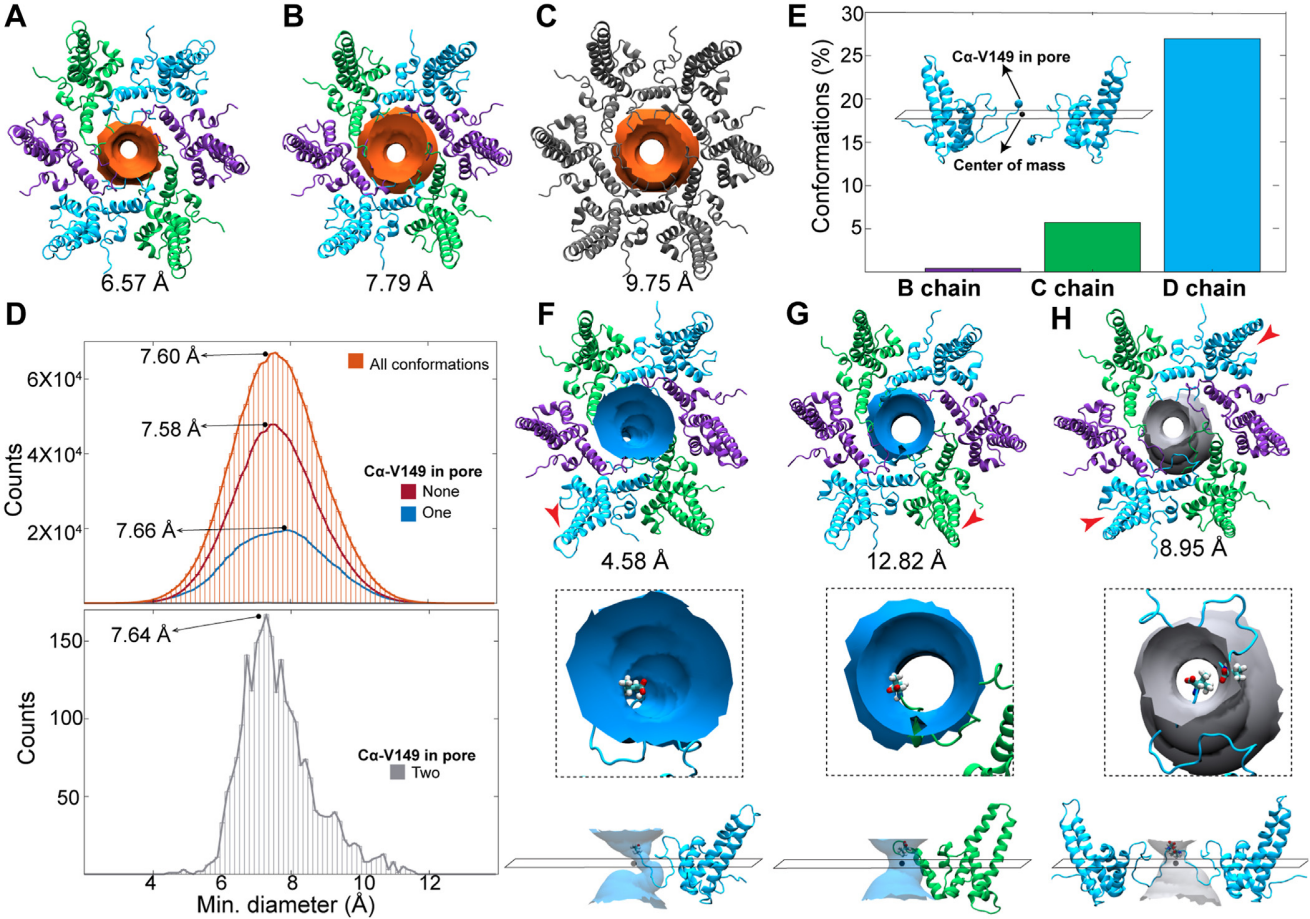
**MD simulations reveal equilibrium behavior of hexameric pore**. *A*–*C*, a surface showing the minimum pore diameter of (*A*) an empty capsid hexamer (from PDB 2G33), (*B*) a RNA-filled capsid hexamer (from PDB 3J2V), and (*C*) the focused hexamer reconstruction from this study (PDB 8GHS). *D*, distributions of minimum pore diameters measured for 1.5 million hexamer conformers extracted from 1-μs MD simulation of intact HBV capsid: all conformers (*orange*), conformers without Val149 in pore (*red*), conformers with one copy (*blue*), and two copies (*gray*) of Val149 extended through pore bottleneck. Note that our observed values of 9.75 Å for a pore with bound Impβ is within two standard deviations of the mean. *E*, percent of hexamer conformers occupied by quasi-equivalent *B*, *C*, and *D* termini. Note the comparison of an internal CTD with a CTD in the pore (inset). *F*–*H*, exterior and side views of representative conformers exhibiting the (*F*) minimum and (*G*) maximum pore diameters where Val149 of one monomer extended through and (*H*) two monomers extended through. Minimum pore diameter measured with HOLE. The hexamer color scheme is as described in Figure 2. CTD, C-terminal domain; HBV, Hepatitis B virus; MD, molecular dynamics.

We examined the dynamics of the hexameric pore by measuring the minimum diameter of 1.5 million simulation conformers. The pore diameter is 6.57 Å for PDB 2G33 (30), upon which the simulation model was based, and 7.79 Å for PDB 3J2V (28) (Fig. 4, *A* and *B*). In contrast, the focused Impβ-bound hexamer reconstruction has a pore diameter of 9.75 Å (Fig. 4*C*), expanded more than 1.5σ beyond the simulation mean of 7.60 Å (Fig. 4*D*, orange). Although this larger value falls in the tail of the diameter distribution, it remains within normal equilibrium fluctuation, indicating that significant structural changes to the pore were not induced by or required for Impβ binding. On the other hand, smaller pore dimensions observed during simulation were typically associated with transient asymmetric distortion of the hexamer (Fig. S7).

### Dilation of the hexameric pore is not required for CTD exit

Although the simulated capsid model lacks the CTD, the dynamics of the terminal residue incorporated into the simulation, Val149, allows us to examine the relationship between CTD exit and pore diameter. In full-length Cp183, Val149 is part of the linker domain (31, 32). In 33% of hexamer conformers sampled during simulation, Val149 of a constituent monomer was observed to be extended into the tunnel of the pore, beyond the bottleneck of the pore’s minimum diameter. This behavior was primarily observed for D monomers (Fig. 4*E*) and had no discernable correlation with pore diameter, as central pores occupied by Val149 still sampled the full range of equilibrium dimensions (Fig. 4*D*, blue). Representative snapshots of hexamers exhibiting the extremes of small and large pore diameters, respectively, are shown in Figure 4, *F* and *G*; inset and side-view illustrate extension of Val149 through the pore tunnel. Notably, C termini need not be extended through the center of the pore but may interact with the tunnel walls. On rare occasion (<0.2% of conformers), the simulation captured a hexameric pore occupied by two termini (Fig. 4*H*), which in all cases were D monomers. Although sampling of this state was limited, the asymmetric diameter distribution (Fig. 4*D*, gray) indicates that it occurs more commonly when pores adopt average-to-large dimensions. Reported asymmetric distortion of hexamers (Fig. S7), which entails elongation along the axis of D monomers, could relate to the ability of two D termini to penetrate the pore simultaneously. We note that the transition from the hexagonal large pore to the elongate small pore conformation is reminiscent of the structural transition of quasi-6-folds associated with capsid expansion in bacteriophages (33, 34).

### CTDs of different quasi-equivalent Cp183 monomers exit the central pore

The hexamer density maps were examined to locate where the CTDs exited the capsid to interact with host importins. Based on its central location under the cloud of Impβ density, the central pore of the hexamer is a reasonable location for an exiting CTD. Alternatively, a CTD could extrude out of the trimeric 3-fold and quasi-3-fold pores (Fig. 1) or even at dimer–dimer interfaces during capsid breathing (26).

**Figure 5:**
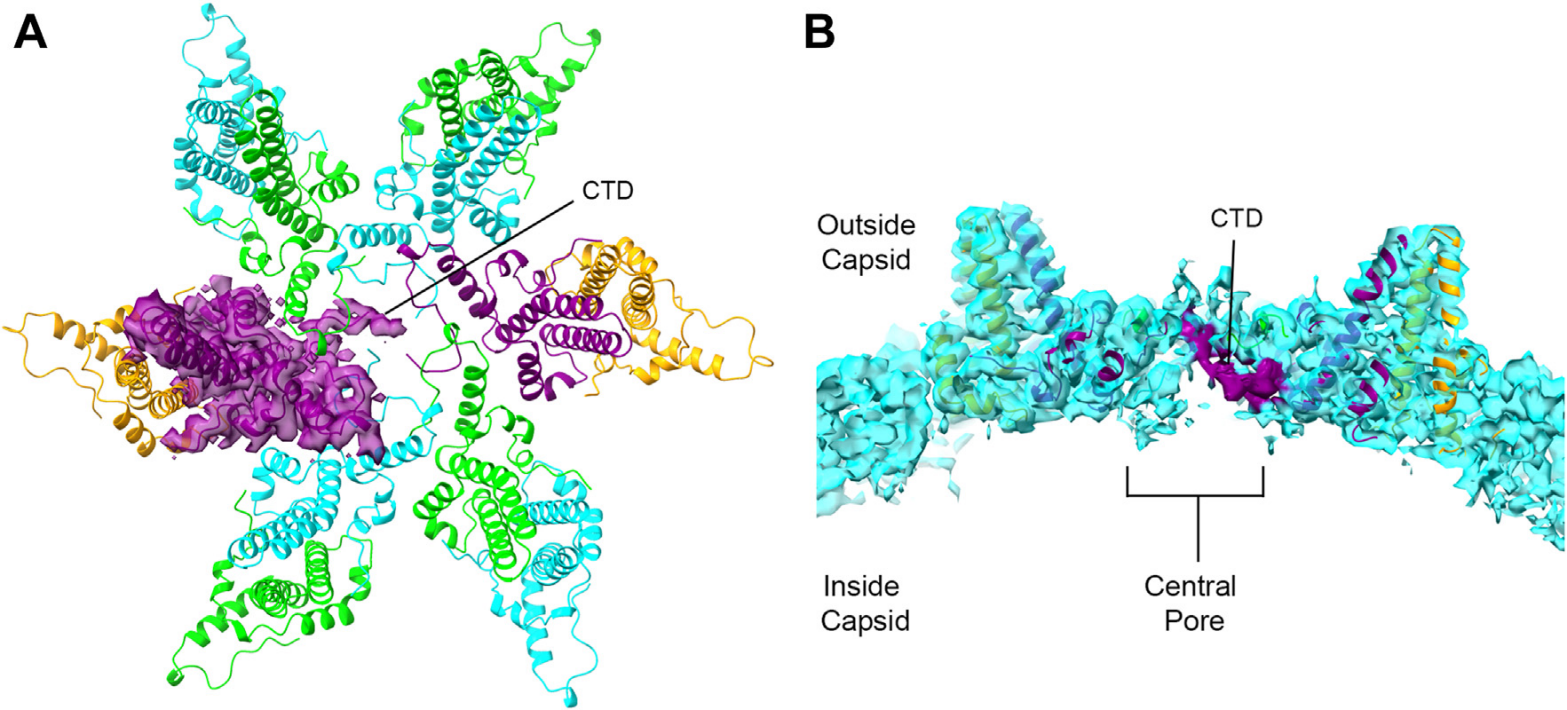
**CTD density from the B and D monomers extends toward the central pore, while CTDs of C monomers extend away from the pore.***A* and *B*, in both the hexamer focused reconstruction (*A*) and the asymmetric capsid reconstruction (*B*), the CTDs of the B and D monomers extend toward the pore, while those of the C monomers extend away from the pore. *A*, a view of the quasi-6-fold from the exterior, showing density for only 1 B (*purple*) subunit for clarity. The B CTD extending to residue 151 is clearly visible in the central pore. The cartoon diagram of the D subunit (*cyan*) shows a similar extension. Conversely, the CTD of the C (*green*) subunit is hidden under the protein. *B*, when viewed from side, CTD density (highlighted in *purple*) extends through the central pore. In this case the density is from the asymmetric reconstruction of whole capsid. Density extending beyond the capsid cannot be assigned unambiguously to either of the 2 B or two D subunits. The *color* scheme is as described in Figure 2. CTD, C-terminal domain.

In both the focused reconstruction of a hexamer and the C1 capsid reconstruction, the map visualized at a contour level of 1σ showed CTD densities adopting different conformations (Fig. 5). The CTDs of the B and D monomers extended toward the central pore, while those of the C monomers extended away from the pore (Fig. 5*A*). In the focused hexamer, the CTD density of 1 B monomer allowed the model to be extended by five alanines beyond Thr146, the last residue with a defined side chain. With the C terminus extended to residue 151, the B CTD was long enough to exit the capsid. In the capsid map, in the majority of the hexamers, the strongest density for exiting CTDs belonged to the D monomers, though both B and D appeared able to exit the pore.

We were able to visualize density, presumably CTDs, extending through the central pore in both hexamer and capsid maps (Fig. 5*B*). It is unclear from the hexamer or capsid reconstructions if there is a preference between the B or D monomers. Extrapolation of our MD analysis suggests there is a preference for escape of D. Furthermore, the pore is large enough for two CTDs to fit simultaneously according to simulation, but two cannot be differentiated in our map. As a final caution, it is possible the differences observed with CTD densities from the B and D monomers are the result of differences in Impβ ratios or the data processing approach.

## Discussion

We observed that empty HBV capsids may adapt to binding Impβ by local quaternary conformational changes. This expansion of the hexamer pore diameter is within normal capsid dynamics but does not represent a common conformation (Fig. 4, (26)). Cryo-EM reconstructions show that the CTDs of the B and D monomers were observed protruding through the hexamer central pore to interact with Impβ. The reconstruction suggests a basis for preferred exposure of quasi-equivalent CTDs, consistent with the multiphase kinetics of CTD sensitivity to proteases (2, 4). We consider how the environment of the CTD and the structure of the capsid contribute to CTD egress.

What is the environment of the CTDs and how does that modulate CTD exposure? Strikingly, in reconstructions of HBV cores filled with nucleic acid, there is a wide gap between the protein shell and the nucleic acid density implying that the CTDs are disordered (3, 11, 28, 35). A recent NMR examination of RNA-filled capsids showed that CTDs are in an irregular and fluctuating environment (36). These results suggest that the CTDs form a liquid–liquid phase separation (37). In an empty Cp183 capsid, the likely driving force for CTD egress is electrostatic repulsion, *i.e.*, to decrease the density of internal charge. *In vivo*, the CTDs of empty capsids are enriched in phosphorylations (13, 38) which would enhance CTD–CTD interaction without necessarily imposing order; CTD exposure is steeply attenuated by phospho-mimetic CTD mutations (2). In ssRNA-filled capsids, where the nucleic acid is flexible and includes enough nucleotide to bind all CTDs (an approximate 1:1 match), CTDs are highly resistant to egress (7, 14, 27), though still an irregular fluctuating liquid (36). The mature virus presents a unique environment and requires CTD egress to initiate infection. In mature HBV, CTDs are largely unphosphorylated, and the interior is crowded with the circular, largely double stranded DNA genome. Because dsDNA is structurally constrained within a capsid (persistence length of dsDNA is 500 Å *versus* a maximum internal diameter of 250 Å for capsid), we speculate that the packaging of the DNA leaves a subset of CTDs free to exit the capsid.

We propose that the plasticity and quasi-equivalent nature of the capsid influence CTD exposure. MD simulation of the T = 4 capsid demonstrated that HBV could subtly compress or elongate under equilibrium conditions (26). It can be argued that this asymmetric distortion of the capsid may selectively allow CTDs of certain regions to escape the capsid lumen. In the same study, the hexameric regions of the capsid lattice were found to be more flexible than the pentameric regions (26). In addition, dimers have been shown to exhibit high flexibility in helix 5 region (39), which could influence the distal linker and C-terminal regions. These structural changes qualitatively correlate with structural changes we observed in both AB and CD dimers (Fig. 3).

A second feature that is expected to affect exit of the CTD from the capsid is the Cp sequence. Preceding the CTD, residues 140 to 149 comprise a linker between the assembly domain and CTD. Mutations or truncations in the linker region have been shown to impair RNA packaging and viral DNA synthesis (32). The linker also modulates assembly of empty capsids (31, 40). These observations point to the linker region’s influence on the dynamics of the capsid and the CTDs. Furthermore, we observe that it is the linker that occupies the pore when the entire CTD is extended. However, our MD analysis found no correlation of the pore diameter with presence of the linker inside the tunnel of the pore, indicating that pore expansion is not required for CTD egress which may reflect that the capsid is a large and interconnected molecular machine.

Finally, we observed that the diameter of the pore is relatively narrow. It is wide enough for one, or very rarely two, peptide chains to extend through, but would be crowded by a hairpin and would have to rupture to accommodate additional CTDs. In MD simulations, the narrowest pore diameter is ∼4.5 Å and the widest is ∼13 Å. Egress of Val149 was possible across the full range of pore dimensions. Yet, we have previously observed as many as 90 Impβ bound to a capsid, with many internalized (27). Thus, capsid rupture followed by reannealing apparently is not an unusual event and speaks to the flexibility of the HBV capsid.

Impβ traps a quaternary structure that has not been seen in other HBV capsid structures (23, 28, 30, 41, 42). However, MD simulations show that a full capsid can undergo tremendous structural variation, with structural displacements up to 8 Å and a capsid that is never truly symmetric at any point in the simulation (26). Compared to the equilibrium range of accessible pore dimensions sampled by MD, the size of the quasi-6-fold pore observed in our hexamer reconstruction is unusually expanded (Figs. 1 and 4). Although CTD egress does not appear to require dilation of the pore, exposure of these termini are relatively rare phenomena, as proteolytic sensitivity experiments reveal that cleavage occurs with a half time of minutes (2, 4). Interaction of multiple copies of the CTD within the capsid interior may lead to crowding and competition for pore entry. Consistent with proteolytic studies is our observation that not all quasi-equivalent CTDs have equal access to the pore: B and D CTDs both extend into the pore. This observation has implications relating CTD organization to capsid stability.

The HBV capsid is a complex ensemble of Cp subunits, which can play diverse roles depending on their state. The capsid can test its environment by exposing CTDs, and it can respond to its environment by conformational change. We have shown that not all CTDs have equal access to the capsid exterior. We have also shown that CTD exposure can lead to a local distortion of a quasi-6-fold vertex, which if not evenly distributed over the capsid has potential to result in asymmetric distorted particles observed in cryo-EM data (41, 42). Conformational change can be transduced across the capsid, resulting in allosteric effects up to and including capsid rupture. Capsid dynamics and the ability to respond to environment are vital to the ability of the virus to bind importins, traffic within a cell, and to eventually uncoat to release its genome to the nucleus.

## Experimental procedures

### Protein purification and preparation of Impβ-bound HBV capsids

Expression and purification of *E. coli* RNA-filled Cp183 capsids and Impβ were purified as described previously (2, 27). *In vitro* assembled empty Cp183 capsids were prepared as previously described (4). In brief, *E. coli* RNA-filled Cp183 capsids were disassembled in 1.5 M GuHCl, and Cp183 dimers were purified *via* size-exclusion chromatography. Cp183 dimers were reassembled into empty capsids by dialysis with high NaCl-containing buffer. After, capsids were separated from free dimers *via* size-exclusion chromatography. To assemble Impβ-bound capsids, Impβ was first buffered exchanged into 0.5 M NaCl, 20 mM Tris-HCl pH 7.5, and 10 mM DTT by running the protein through a Superose 6 column. Empty capsids, typically at 11.9 μM with respect to the amount of Cp183 dimer (or about 0.01 μM capsid if 100% of capsids had T = 4 symmetry), and Impβ were mixed in a 1:80 or 1:205 ratio (capsid:Impβ). Then, the mixture was dialyzed overnight against 100 volumes of 0.15 M NaCl, 20 mM Tris-HCl pH 7.5, and 10 mM DTT at 4 °C. After the overnight dialysis, the sample was immediately prepared for cryo-EM.

### Cryo-EM reconstruction and structural analysis

To prepare grids for both the low and high Impβ samples, 4 μl of Impβ-bound capsids were applied four times (to enrich for particles) to glow-discharged 300 mesh Quantifoil R2/2 holey carbon grids. Sample grids were vitrified using a Thermo Scientific Vitrobot.

For the sample with the low ratio of empty Cp183 capsid to Impβ (1:80), cryo-EM images were collected using a Titan Krios G3i (Thermo Fisher Scientific) operated at 300 kV, equipped with a Gatan BioQuantum K3 camera with the energy slit opened at 30 eV. The data acquisition was performed using EPU software (Thermo Fisher Scientific). Images were collected using counted super-resolution mode at a nominal magnification of 64,000×, which has a pixel size of 0.7 Å at the final image. The total dose for each image was aimed at 30 e^-^ per Å^2^ and the dose fraction was set at 1 e^-^ per Å^2^ per frame. A total of 3978 images selected after motion correction and contrast transfer function evaluation were used for data processing. Particles were semi-manually picked using e2boxer.py from EMAN2 (43) and the coordinates were imported into Relion (v3.1.3) (44) for extraction. Multiple runs of 2D classification were used to further discard ice contamination or background density. Approximately 442,227 particles were selected for T = 4 sized particles. The initial model was built *de novo* using C1 symmetry in Relion. The 3D structure refinement started asymmetrically (using C1 symmetry) at a high binned sampling rate (bin = 8) and gradually moved up to bin = 2 (pixel size is 1.4 Å) for final data analysis. Throughout this process, no icosahedral symmetry was enforced. After contrast transfer function refinement, particle polishing, and post processing (which included an automatic estimation of the B-factor and a lowest resolution setting for auto-B fit), the 3D reconstruction converged to 3.8 Å, as gauged by the gold-standard resolution estimation (Fourier shell correlation at 0.143) implemented in Relion.

To further analyze the CTD exposure, focused classification was performed (42). In brief, a spherical binary mask capable of accommodating six dimers centered at 2-fold vertices (*i.e.*, the quasi-6-fold) was created by using the segmentation function in ChimeraX. The location of the mask was selected based on our 3D reconstruction, which indicated Impβ binding to the capsid at the 2-fold position. This mask was moved to all 30 2-fold locations on the T = 4 reconstruction. Given that our model was refined using C1 symmetry, we could directly subject the structures of the capsid protein at this particular location to 3D classification in Relion, omitting further image alignment using K = 8 (representing the number of classes) and tau2_fudge=50 (a regularization parameter used during 3D classification to enhance the signal-to-noise ratio). The resulting cryo-EM maps were rigid-body fit with a molecular model (PDB 3J2V) to evaluate the exposure of the CTD density using ChimeraX (45).

For the high capsid:Impβ sample (1:205), grids were imaged with a Talos Artica microscope (Thermo Fisher Scientific), operated at 200 kV and equipped with a Falcon III direct electron detector. A total of 851 micrographs were collected at a nominal magnification of 150,000 (pixel size 0.97 Å) and motion corrected using Relion. The contrast transfer function parameter was estimated with ctffind4. A total of 31,166 HBV particles were manually and autopicked using e2boxer.py from EMAN2. Using Relion, the extracted particles underwent multiple rounds of reference-free 2D classification to enrich for classes with high quality T = 4 capsids. Eighteen classes (29,006 particles) were used to generate an initial *de novo* model with C1 symmetry, in which the data were binned (bin = 2; pixel size 1.94 Å). This model was used as a reference map for 3D refinement with I2 symmetry until no further resolution improvement was observed. This process resulted in a final resolution of 4.2 Å. For the focused classification, the orientations of each particle were expended to encompass all 60 icosahedral equivalent locations, using relion_particle_symmetry_expand command with I2 symmetry. The same binary mask that accommodates six dimers was used to extract a total of 1,730,160 subparticles at the quasi-6-fold locations (referred as hexamers). These particles were used to generate a new hexamer model with C1 symmetry, subsequently serving as a reference map for 3D classification.

The resulting hexamer models were categorized based on their right- or left-handedness, with 566,641 and 588,251 particles, respectively. Notably, the right-handed model aligns with previously published cryo-EM and PDB structures. Accordingly, the left-handed particles were reoriented using xflip_particles_star.py from starpy (https://github.com/fuzikt/starpy) and combined with the right-handed particles. Finally, the consolidated particles were subjected to 3D refinement and postprocessing, which included an automatic estimation of the B-factor and the setting of the lowest resolution for auto-B fit to 10, using the default parameters in Relion.

A hexamer model was flexibly fit into the hexamer maps using Alphafold, ISOLDE, and Phenix (46, 47, 48, 49). In brief, the AB and CD asymmetric unit (using the protein sequences from pdb: 3J2V) was generated using Alphafold to produce a model with no geometry outliers. ISOLDE was used to fit the carbon backbone of the model into the hexamer map and fix initial outliers; the resulting model was used to build in the rest of the hexamer model. The hexamer model was further refined using Phenix real space refinement with no symmetry constraints imposed. Coot was used to add and fit alanine residues into the CTD densities of one of the hexamer maps (50).

### MD simulation analysis

Computational analysis was based on the trajectory from a previously reported 1-μs atomistic MD simulation of the intact HBV capsid (26). The trajectory was deconstructed into 1.5 million conformations of the capsid hexamer. The principal axes of each capsid hexamer were aligned to the Cartesian axes using the Orient package in VMD (51), such that the quasi-6-fold pore was positioned along the Z-axis. The minimum pore diameter of each hexamer conformation was measured using the HOLE algorithm, as implemented in the MDAnalysis package (52, 53). The cvect [0 0 1] parameter in the hole2.HoleAnalysis module was defined to restrain the pore search to the Z-axis. The random seed was fixed for reproducibility. All atoms from residues 1 to 142 were selected to perform the calculation. Analysis was carried out on the DARWIN supercomputer using VMD compiled with MPI support.

To track translocation of C termini through the minimum diameter of the central pore, the center of mass of each hexamer was measured based on the Cα atoms. The hexamer conformations were translated to place the center of mass at the Cartesian origin. We defined a reference plane in the XY direction that intersects each hexamer conformation through its center of mass. Monomer termini were classified as being extended through the pore bottleneck if the position of the Val149 Cα had a positive sign, indicating its location above the reference plane.

## Data availability

Final hexamer molecular coordinates are deposited in the PDB with accession number 8GHS and corresponding EMDB entry ID EMD-40049. The C1 map of a complete particles has EMDB entry ID EMD-40048. The data derived from MD simulations that support the findings of this study are available from J.A.H.-P (jhadden@udel.edu) upon request.

## Supporting information

This article contains supporting information.

## Conflict of interest

A. Z. has interests in Assembly BioSciences and Door Pharmaceuticals, companies that are developing HBV-specific antivirals. The other authors have no conflict of interest with the contents of this article.
