# Supplementary material for: Structure of the Hepatitis B virus capsid quasi-6-fold with a trapped C-terminal domain reveals capsid movements associated with domain exit
Source: J Biol Chem. 2023 Jul 28;299(9):105104. doi: 10.1016/j.jbc.2023.105104 (PMC10463254; doi:10.1016/j.jbc.2023.105104)
Supplement: Supporting Figures S1–S7 [file mmc1.pdf]

Supporting Information Figures

Structure of the Hepatitis B Virus capsid quasi-sixfold with a trapped C-terminal domain reveals capsid movements associated with domain exit

Christine Kim[1], Christopher J. Schlicksup[1,2], Carolina Pérez-Segura[3], Jodi A. Hadden-Perilla[3], Joseph Che-Yen Wang[4]*, Adam Zlotnick[1]*

* Co-contributing authors

Supporting Figures

Figure S1. Schematic for sample preparation and image reconstruction strategies.
Figure S2. 2D classification of capsids shows that most particles are spherical and externally decorated with Impβ.
Figure S3. An Impβ-capsid complex reconstructed with C1 symmetry showed uneven occupancy of the quasi-sixfold sites.
Figure S4. Focused reconstructions of a quasi-sixfold hexamer of dimers and surrounding dimers.
Figure S5. Superposition of the apo and flexibly-fit Cp monomers indicate that they retain their tertiary structures.
Figure S6. Superposition of a flexibly fit hexamer from an Impβ-decorated capsid onto a hexamer from a Cp183 capsid without Impβ.
Figure S7. Distorted and symmetric hexamers.

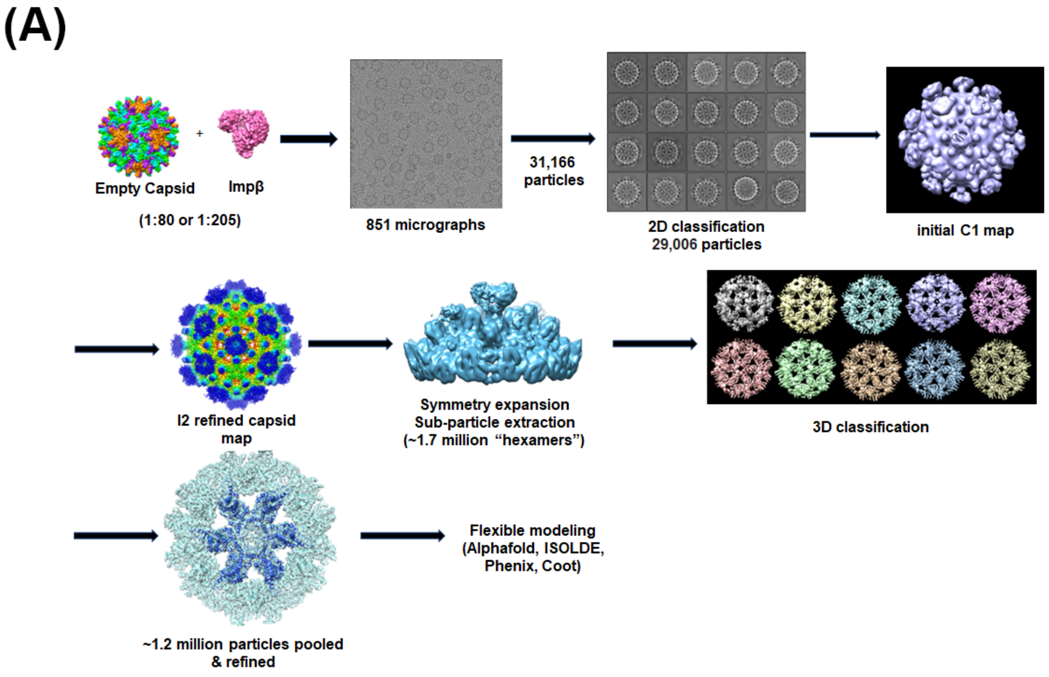

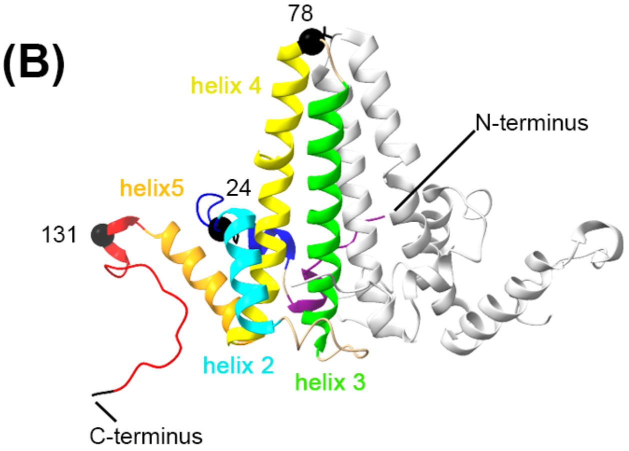

Figure S1. Schematic for sample preparation and image reconstruction strategies. (A) Two strategies were taken to solve the structure of the Impβ-bound capsid complex: an asymmetric reconstruction of a whole capsid and a focused reconstruction of quasi-sixfold. For the asymmetric reconstruction (top row only), capsids at 11.9 µM Cp183 dimer were mixed with 8.0 µM of Impβ at a ratio of 1:80 capsid:Impβ to minimize damage to the capsid. The micrographs collected from these sample were used for capsid reconstruction with C1 symmetry. In the second strategy, focused reconstruction we predicted that we would be able to reject damaged hexamers and thus used a higher concentration of Impβ to maximize occupancy of the quasi-sixfold symmetry axis, mixing capsids (11.9 µM Cp183 dimer) with Impβ (20.3 µM) resulting in a ratio of 1:205. The resulting micrographs were processed to generate capsid structures with icosahedral symmetry (I2). The I2 particle coordinates were symmetry expanded to identify individual quasi-sixfold hexamers which were then subjected to reconstruction, 3D classification, selection, and refinement. (B) A dimer with one subunit colored and labeled to indicate

the N-terminus (purple) and C-terminus (red), selected residues, and helices. Helix 1 (blue) is in back and partially obscured. The $C\alpha$ atoms of selected residues are represented as black spheres.

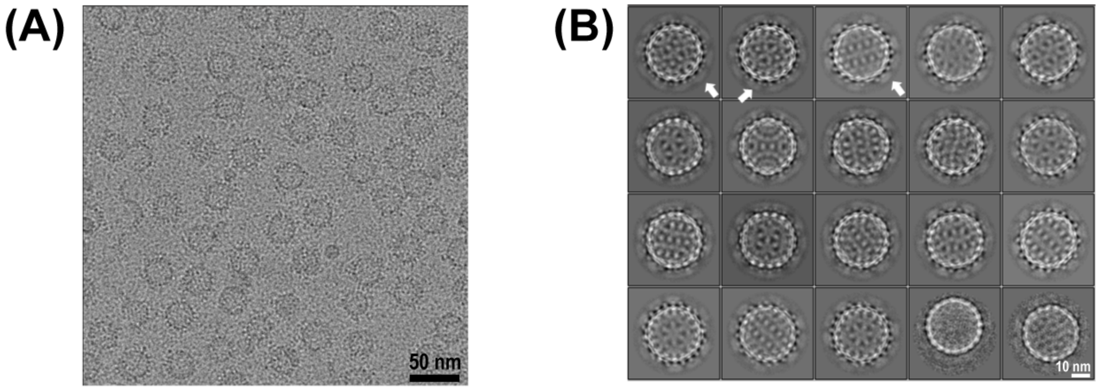

Figure S2. 2D classification of capsids shows that most particles are spherical and externally decorated with Impβ. (A) A representative cryo-electron micrograph shows Impβ-bound capsids (high Impβ sample). (B) After several rounds of 2D class averaging to enrich for intact T=4 capsids, a final round of 2D classification produced 20 classes from 27,443 particles, all containing spherical capsid morphology (classes are in descending order of population). 18 of the 20 classes exhibit funnel-like density decorating the capsid exterior, indicating bound Impβ (arrows point to some representative examples). Note that all of these capsids appear to be empty or “clear”.

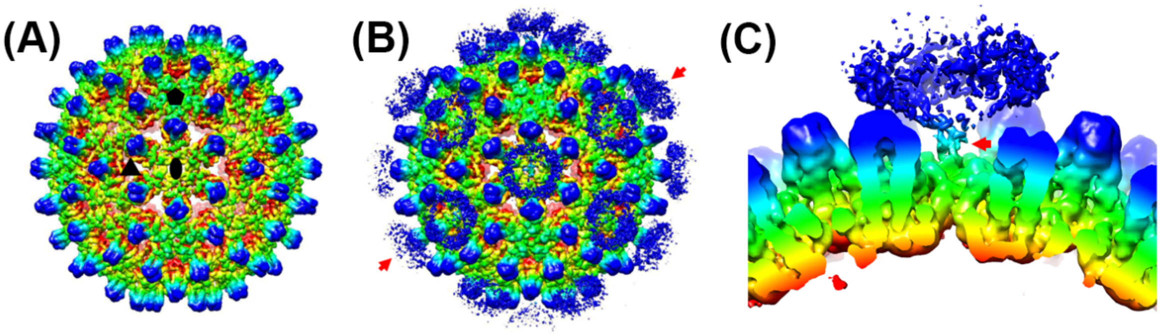

Figure S3. An Impβ-capsid complex reconstructed with C1 symmetry showed uneven occupancy of the quasi-sixfold sites. From the 1:80 ratio sample, T=4 particles were picked and processed to generate a capsid reconstruction with no symmetry imposed to 4.1 Å resolution. (A) Despite no application of symmetry, the capsid density map (contoured at 2.8σ to show only strong density) shows the expected T=4 geometry with no obvious defects. A set of symmetry operators are shown on the figure. (B) At a lower contour level (1σ), we observed rings of diffuse density above the quasi-sixfold vertices. These rings indicate the presence of bound Impβ. Consistent with previous findings, the Impβ densities exhibited heterogenous thickness at different quasi-sixfold sites, suggesting that all sites are not equally occupied. The left-most arrow points to an area of low Impβ density, while the right-most arrow shows an area of high Impβ density. (C) A closer examination of the quasi-six-fold revealed a “rope-like” CTD density (indicated by the arrow) emerge from the central pore and extend into the Impβ density (shown as a cross-section of the capsid; density map was contoured at 1σ).

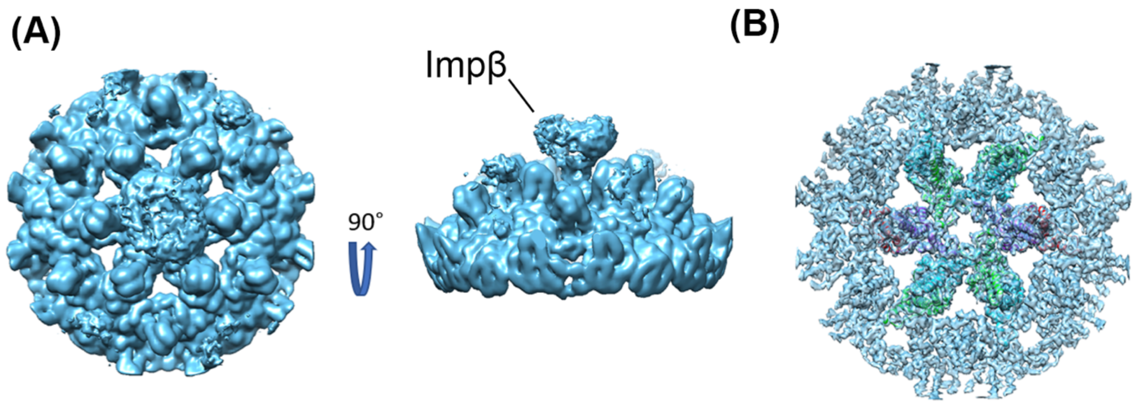

Figure S4. Focused reconstructions of a quasi-sixfold hexamer of dimers and surrounding dimers. Quasi-sixfold hexamers were extracted from a symmetry-expanded I2 reconstruction. (A) The initial hexamer reconstruction shows the Impβ density emerging from the center and extending out into a funnel-like shape. (B) After 3D classification of data used for the reconstruction in panel A, the classes with the strongest and most regular density were pooled and used as the basis of this 4.4 Å structure. The A, B, C, and D monomers are labeled in orange, purple, green, and cyan respectively.

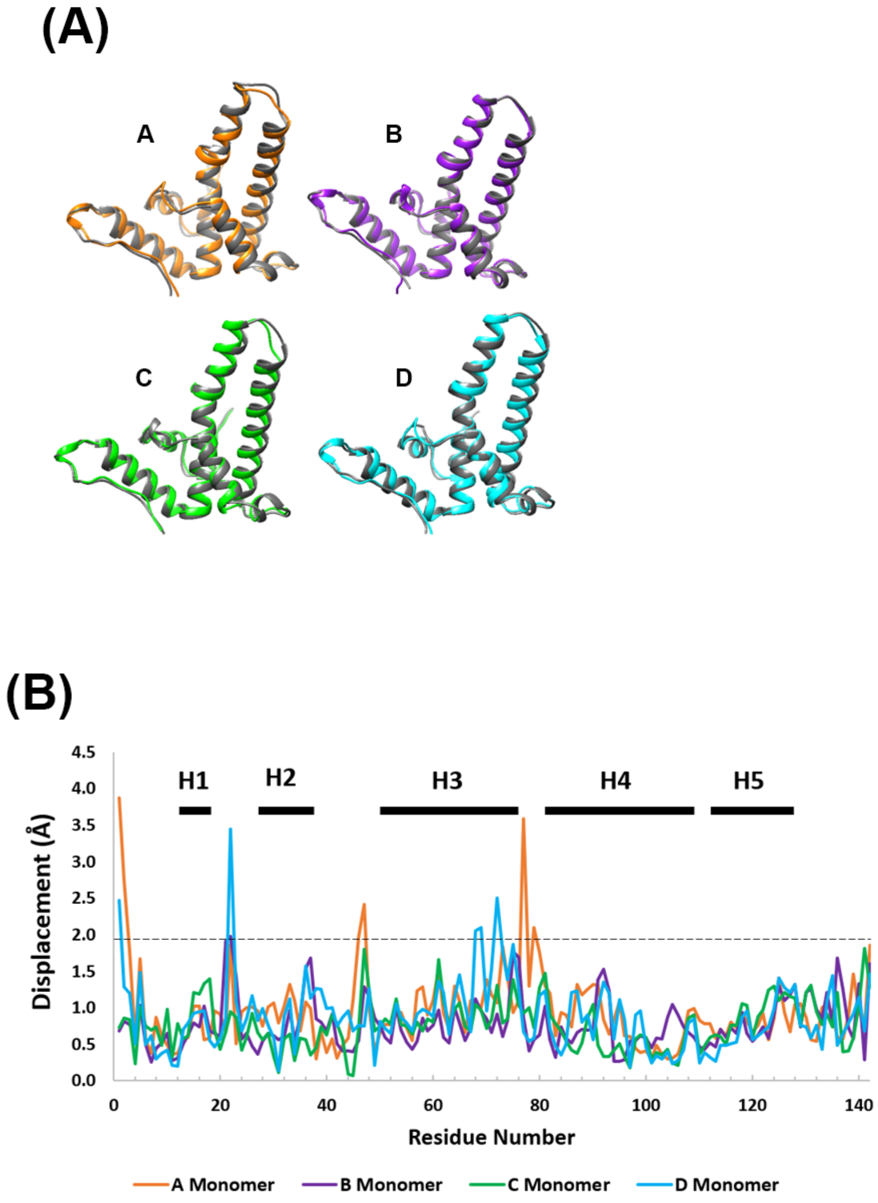

Figure S5. Superposition of the apo and flexibly-fit Cp monomers indicate that they retain their tertiary structures. (A) Cp models (gray) were flexibly fit into the hexamer density map. The A, B, C, and D monomer models (orange, purple, green, and cyan respectively) from a Cp183 capsid (pdb: 3J2V) without Impβ were superpositioned onto the respective monomers from the hexamer model. The calculated RMSD for the A, B, C, and D monomers are 1.09, 0.86, 0.84, and 1.0 Å. (B) The α-carbon displacement for each Cp monomer shows few large dislocations. In the A and D monomers, carbon-α displacement >2 Å were observed in few residues, most near the highly flexible spike tip. Black bars indicate where helices 1-5 are located within the Cp sequence (compare to Fig 2B).

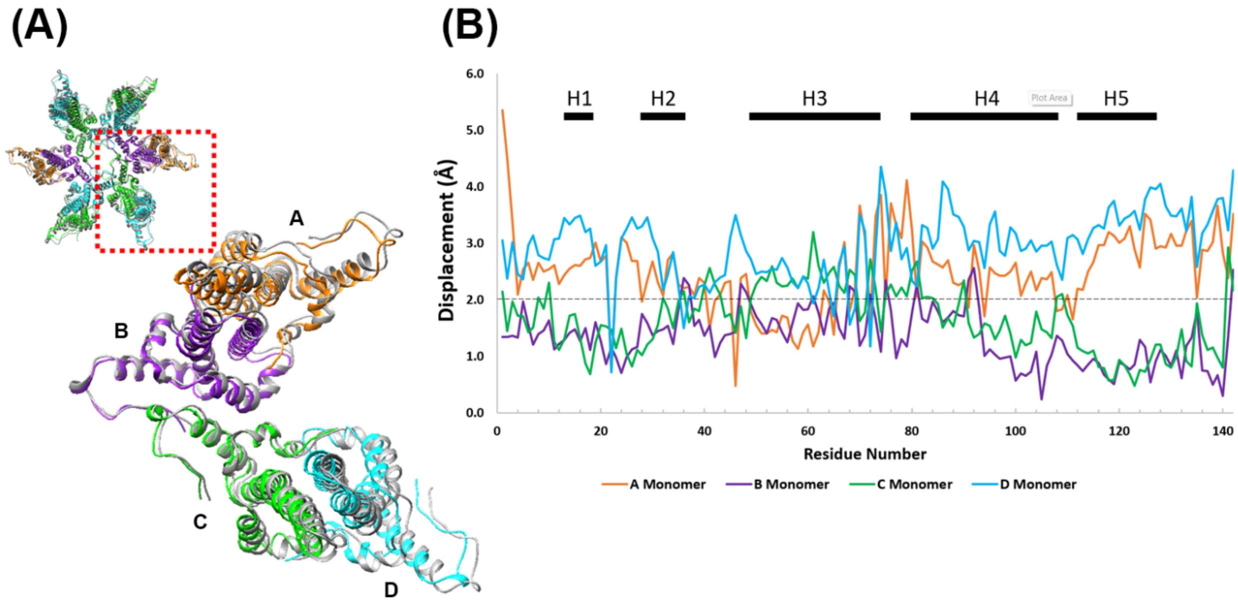

Figure S6. Superposition of a flexibly fit hexamer from an Impβ-decorated capsid onto a hexamer from a Cp183 capsid without Impβ. (Left panel) The model from the focused quasi-sixfold reconstruction (grey) was superposed on an apo-hexamer from an RNA-filled capsid (pdb: 3J2V) as shown in Figure 2. The icosahedral asymmetric unit consists of two dimers; the inset shows a quasi-sixfold with the asymmetric unit boxed in red to provide structural context. (B) The α-carbon displacement for each Cp monomer shows a systematic dislocation. Note that in this diagram we only show the absolute value of the displacement, which may be positive or negative. The minimum displacement for the super position is for the B and C subunits around residue 140, at the loop at the end of helix 5. Subunits B and C show maximal displacement for residues 40-90 at the dimer interface. Conversely subunits A and D have maximal displacement around residue 140 and minimum displacement for residues 35-90.

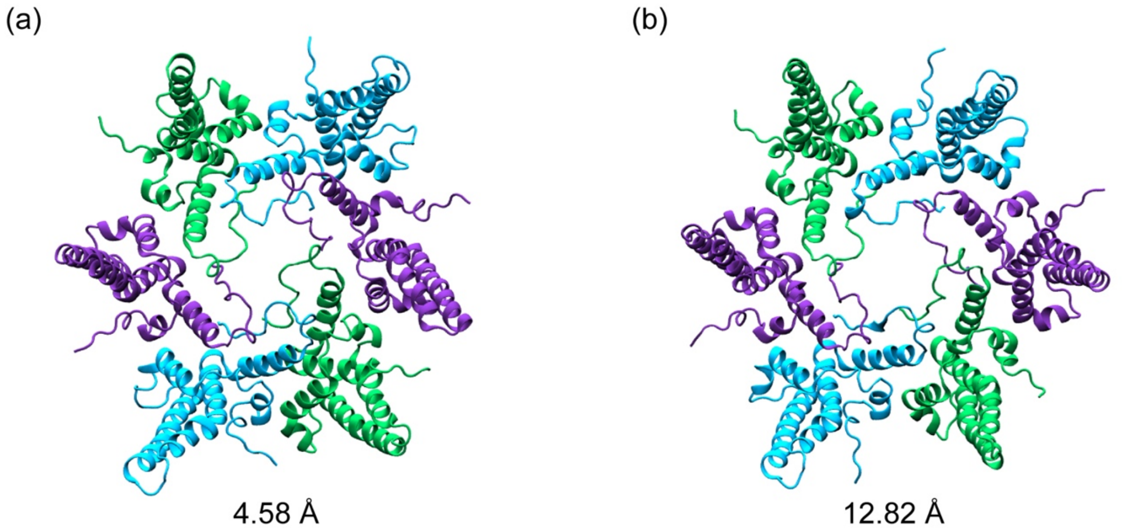

Figure S7. Distorted and symmetric hexamers. Small central pore diameters are attributed to asymmetric distortion of hexamers (a) compared with the relatively symmetric shape of hexamers with enlarged pores (b). The greatest difference in pore dimension is along a horizontal axis.
